# *Loose Panicle1* encoding a novel WRKY transcription factor, regulates panicle development, stem elongation, and seed size in foxtail millet [*Setaria italica* (L.) P. Beauv.]

**DOI:** 10.1371/journal.pone.0178730

**Published:** 2017-06-01

**Authors:** Jishan Xiang, Sha Tang, Hui Zhi, Guanqing Jia, Huajun Wang, Xianmin Diao

**Affiliations:** 1 Gansu Provincial Key Lab of Aridland Crop Science/Gansu Key Lab of Crop Improvement & Germplasm Enhancement/College of Agronomy, Gansu Agricultural University, Lanzhou, People’s Republic of China; 2 Institute of Crop Sciences, Chinese Academy of Agricultural Sciences, Beijing, People’s Republic of China; 3 Chifeng University, Chifeng, People’s Republic of China; Universidad Miguel Hernández de Elche, SPAIN

## Abstract

Panicle development is an important agronomic trait that aids in determining crop productivity. Foxtail millet and its wild ancestor green foxtail have recently been used as model systems to dissect gene functions. Here, we characterized a recessive mutant of foxtail millet, *loose-panicle 1* (*lp1*), which showed pleiotropic phenotypes, such as a lax primary branching pattern, aberrant branch morphology, semi-dwarfism, and enlarged seed size. The loose panicle phenotype was attributed to increased panicle lengths and decreased primary branch numbers. Map-based cloning, combined with high-throughput sequencing, revealed that *LP1*, which encodes a novel WRKY transcription factor, is responsible for the mutant phenotype. A phylogenetic analysis revealed that LP1 belongs to the Group I WRKY subfamily, which possesses two WRKY domains (WRKY I and II). A single G-to-A transition in the fifth intron of *LP1* resulted in three disorganized splicing events in mutant plants. For each of these aberrant splice variants, the normal C2H2 motif in the WRKY II domain was completely disrupted, resulting in a loss-of-function mutation. LP1 mRNA was expressed in all of the tissues examined, with higher expression levels observed in inflorescences, roots, and seeds at the grain-filling stage. A subcellular localization analysis showed that LP1 predominantly accumulated in the nucleus, which confirmed its role as a transcriptional regulator. This study provides novel insights into the roles of WRKY proteins in regulating reproductive organ development in plants and may help to develop molecular markers associated with crop yields.

## Introduction

Foxtail millet [*Setaria italica* (L.) P. Beauv.] and green foxtail (*S*. *viridis*; the wild ancestor of foxtail millet), owing to their unique growing characteristics and small genomes, have emerged as model systems for studying genomics and genetics, C_4_ photosynthesis and stress biology [1–4]. In recent years, our group has used *S*. *italica* as a model for gene mapping and functional genomic studies [5–7]. We used an *S*. *italica* variety with an available genome sequence, ‘Yugu1’, as the material for a large-scale ethyl methylsulfone (EMS)-induced mutant library. We identified an improved variety, ‘SSR41’, which has a similar flowering time and a high level of genetic polymorphism with ‘Yugu1’ [5]. ‘SSR41’ and a mutant originating from ‘Yugu1’ were used as pollen parents to construct mapping populations. Several functional genes, including *SiYGL1* [5], *SiDWARF2* [6], and *SiAGO1b* [7], were discovered recently through map-based cloning. These studies confirmed the potential of *Setaria* spp. to serve as promising models for gene discovery and pathway engineering.

Panicle development is a major component that helps determine crop yield [8]. The molecular mechanisms related to panicle development have aroused wide attention [9]. Approximately 46 genes associated with inflorescence morphogenesis have been cloned and functionally characterized in *Oryza sativa* (http://www.ricedata.cn/). These genes act in various genetic pathways and are mainly involved in regulating the following biological processes: transcriptional regulation (e.g., *LAX1*/*LAX2* [10, 11], *FZP* [12], and *OsMADS15*/*34*/*50* [13]), photoperiods and flowering regulation (e.g., *Ehd1* [14] and *DTH8* [15]), heterotrimeric G proteins (e.g., *DEP1* [16]), and plant hormone regulation (e.g., *GNP1* [17] and *TOB1* [18]). Of these genes, *LAX1*, *LAX2*, *MOC1* [19], and *FZP*, which encode transcription factors and affect patterns of panicle branching, are similar to those investigated in our present study. Most of these transcriptional regulators are highly expressed in axillary meristems and directly regulate their formation, suggesting that transcription factors have extensive and conserved functions in regulating panicle development [11].

The WRKY transcription factor gene family is one of the largest families of transcriptional regulators in plants [20]. The name is derived from its most prominent functional domain, which contains a highly conserved amino acid signature ‘WRKYGQK’ (some specific members have ‘WKKYGNK’ instead). Approximately 74 and 102 WRKY family members have been identified in the model plants *Arabidopsis thaliana* and *O*. *sativa* [20]. Most of the reported studies on WRKY proteins address their involvement in biotic/abiotic stress responses [20, 21], and few characterize their roles in plant growth and seed development. *TTG2* was the first WRKY transcription factor identified as controlling organ development in plant. It is strongly expressed in young leaves, trichomes, and seed coats, and it, together with *TGG1* and *GLABRA2*, controls seed coat morphogenesis and trichome outgrowth [22]. *OsWRKY78* is another example of a WRKY that functions in regulating plant morphology. Both RNA interference (RNAi) and T-DNA insert transgenic lines showed that *OsWRKY78* plays a role in stem elongation and seed development in rice [23]. Other WRKYs regulating seed development (e.g., *SUSIBA2* [24] and *MINI3* [25]), embryogenesis (e.g., *AtWRKY23* [26]), senescence (e.g., *AtWRKY6/22/53* [27]) have also been reported, but there are few reports of WRKYs involved in other developmental processes.

Here, we isolated an EMS-induced *S*. *italica* mutant with loose panicle, semi-dwarfism, and large seed phenotypes. A novel WRKY transcription factor *LP1* was identified as the candidate gene responsible for the mutant phenotypes. Our report reveals a new role for WRKY genes in regulating reproductive organ development in plant.

## Materials and methods

### Plant materials and construction of the mapping population

The *S*. *italica* loose-panicle mutant was isolated from EMS-treated ‘Yugu1’ as described previously [5]. To remove background single nucleotide polymorphisms (SNPs) and produce a steady and homozygous *lp1* mutant line, the mutant was backcrossed with the parental ‘Yugu1’, generating the BC_1_F_2_ population. For map-based cloning, *lp1* was cross-pollinated with the foxtail millet cultivar ‘SSR41’, resulting in the F_2_ mapping population. The leaves of paternal, maternal, heterozygous F_1_, and recessive F_2_ individuals were collected for later use.

### Plant growth and agronomic trait measurements

Plants used in our experiment were grown at Shunyi Station of the Chinese Academy of Agricultural Sciences during growth period from June to October in 2015 (Beijing, China). Ten uniformly developed *lp1* and ‘Yugu1’ plants were collected at the mature stage for agronomic trait investigation. The major agronomic traits including plant height, panicle length, seed size, and 1,000-grain weight were assessed, with 10 biological replications. The investigation and scoring standards used in this study were described previously [28].

### Map-based cloning and candidate gene identification

In total, 234 recessive individuals collected from the *lp1* × ‘SSR41’ F_2_ population were used for gene mapping. DNA samples from leaves were extracted according to a standard CTAB method. For preliminary mapping, equal quantities of 20 DNA samples from recessive individuals were mixed. A bulked segregation analysis was employed to map the candidate gene *LP1* to 41.2–45.5 Mb on chromosome 2 using previously developed SSR markers [28, 29]. For fine mapping, a batch of new insertion–deletion (indel) and dCAPs molecular markers were designed based on the genome sequence of the candidate region (S1 Table). Using these molecular markers for screening the 234 F_2_ recessive individuals, we located the *LP1* gene in a candidate region on chromosome 2.

To identify the candidate gene, we used whole genome resequencing and a MutMap [30] analysis. Equal amounts of DNA samples from 30 recessive individuals of the *lp1* × ‘Yugu1’ BC_1_F_2_ population were mixed and used for DNA library construction. Whole genome resequencing was carried out on the Illumina HiSeq 2500 platform using the 150-bp paired-end strategy. Raw sequencing data obtained for the MutMap analysis has been deposited at EMBL-EBI in the European Nucleotide Archive database under the accession number ERP022965. According to the MutMap method [30], the sequencing reads generated from a DNA pool of 30 BC_1_F_2_ recessive individuals were aligned to the *S*. *italica* reference genome (phytozome.jgi.doe.gov) using BWA software (bio-bwa.sourceforge.net). SNPs were identified using SAMtools software (samtools.sourceforge.net) with default parameters. Low-quality SNPs with read depths less than three for homozygous (five for heterozygous) sites, or a mapping quality value less than 20, were excluded because these SNPs may represent false positives, which would affect gene isolation. The SNP-index value was calculated using a previous report [30]. A sliding window analysis was carried out using the SNP-index values of five consecutive SNPs and one SNP increment. A regression curve was plotted using R scripts (www.r-project.org). All of the SNPs located in the candidate region with index value = 1 were collected for phenotype-relevant SNP identification. To verify the causative SNP, we designed dCAPS markers and screened homozygous recessive individuals from the BC_1_F_2_ population. Only the SNP that co-segregated with all homozygous recessive individuals was regarded as the causal one. The primer pairs used for gene mapping are listed in S1 Table.

### Gene expression analysis and subcellular location experiment

To study the tissue-specific expression of *LP1* in foxtail millet, we collected stems, leaves, roots, nodes, panicles at the booting, heading, and flowering stages, and seeds at the grain-filling stage. RNA extractions and qRT–PCR assays were carried out as described previously [7], with three independent biological replications. The primers used in qRT-PCR are listed in S1 Table.

To test the subcellular localization of LP1 in foxtail millet, we constructed a LP1-GFP fusion protein, and it was transfected into *S*. *italica* mesophyll protoplasts. Detailed methods for vector construction, protoplast isolation and transfection were as previously published [6].

## Results

### The *lp1* mutant attributes the loose panicle phenotype to increased panicle lengths and decreased primary branch numbers

**Fig 1:**
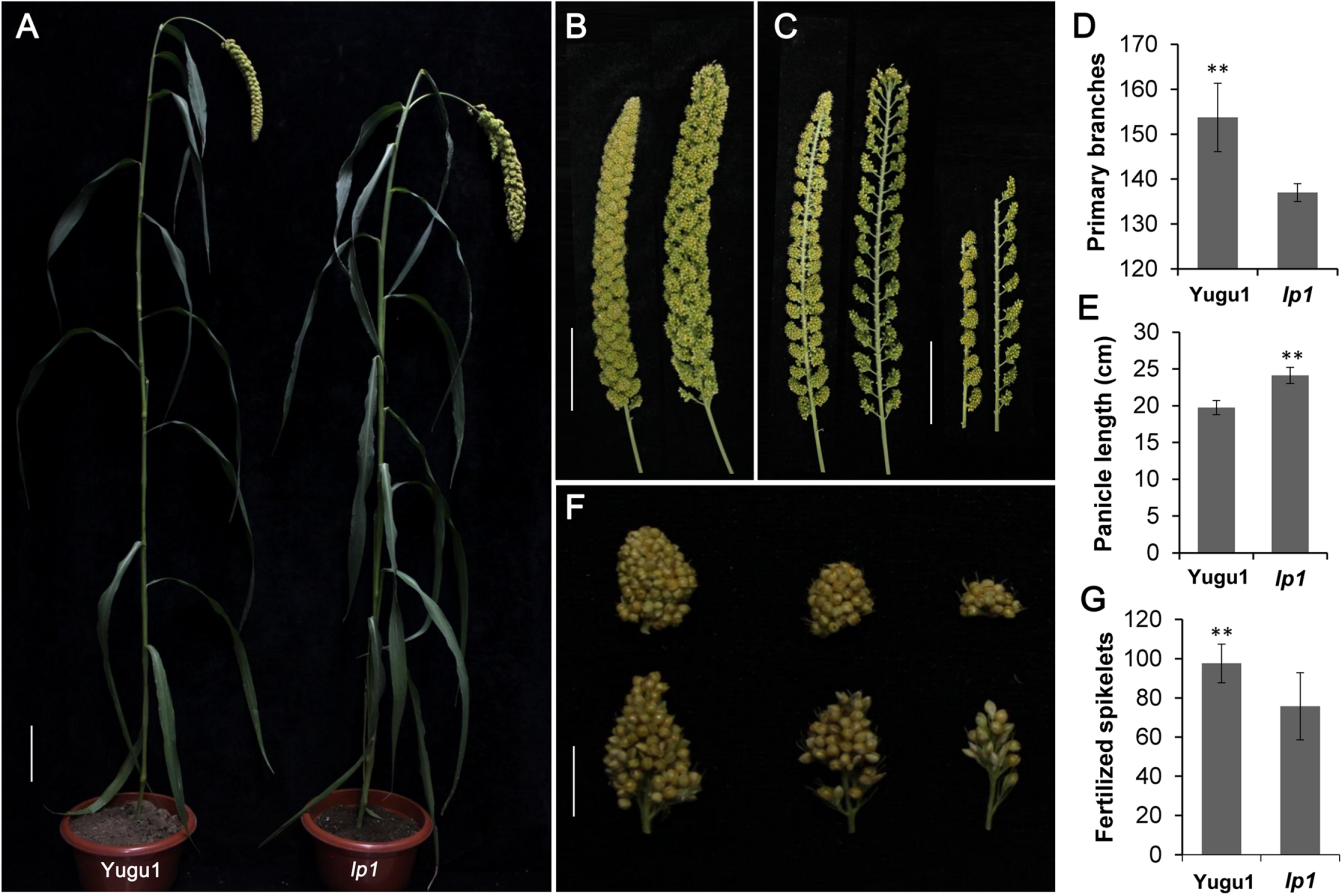
Morphological comparison between the wild-type ‘Yugu1’ and the *lp1* mutant. (A) The general statures of ‘Yugu1’ and *lp1* at the mature stage, bar = 10 cm. (B and C) The panicle morphology of ‘Yugu1’ (left) and that of *lp1* (right), bar = 10 cm. (D, E, and G) Statistics for primary branch number, panicle length, and fertilized spikelets in ‘Yugu1’ and *lp1*. Welch’s two-sample t-test, n = 10 biological replications, asterisks indicate P < 0.01. (F) The primary branch morphology of ‘Yugu1’ (up) and that of *lp1* (down), bar = 1 cm.

The *S*. *italica lp1* mutant was isolated while screening for EMS-induced mutants having abnormal panicle development. The *lp1* mutant exhibited a steady and distinct loose-panicle phenotype compared with the wild-type ‘Yugu1’. As shown in Fig 1A–1E, *lp1* showed a decreased plant stature as panicle size increased. There were ~137 primary branches per panicle in the mutants, compared with ~154 primary branches in wild-type plants. Moreover, the average panicle length of *lp1* was ~22.2% longer than that of ‘Yugu1’. Thus, the loose panicle phenotype of the *lp1* mutant was attributed to the reduction in primary branch numbers and increase in panicle length. In addition, the lengths and widths of primary branches increased in the mutant, while the numbers of fertilized spikelets and seed setting rates significantly decreased (Fig 1F and 1G and Table 1), suggesting an effect of *lp1* in the morphogenesis of primary branches and reproductive growth.

**Table 1:** Morphological traits of *lp1* and wild-type ‘Yugu1’.

	Yugu1	*lp1*	*t* value	*P* value
Plant height (cm)	122.03±3.45	136.00±5.40	-6.54	<0.0001
Main stem diameter (mm)	5.69±0.41	6.25±0.74	-1.98	0.0652
Main stem node number	12.11±0.93	12.67±1.08	-1.22	0.2395
Peduncle length (cm)	28.80±1.37	20.35±0.65	9.62	0.0007
Leaf length (cm)	39.22±1.68	39.12±2.06	0.11	0.9116
Leaf width (cm)	2.72±0.13	2.84±0.17	-1.68	0.1114
Panicle length (cm)	19.74±0.96	24.13±1.11	-5.94	0.0010
Panicle diameter (mm)	21.22±2.61	23.12±2.12	-1.08	0.3141
Panicle weight (g)	20.97±1.34	14.71±1.67	5.89	0.00304
Bristle length (mm)	1.82±0.30	2.26±0.17	-2.70	0.0355
No. of seeds per panicle	6947.60±170.30	3982.00±309.76	4.18	0.0058
Grain weight per panicle (g)	18.95±3.92	12.48±1.57	2.67	0.0371
1000-grain weight (g)	2.72±0.20	3.33±0.14	-4.67	0.0034
No. of primary branches (PB)	153.73±7.63	137.00±2.00	3.62	0.0111
No. of secondary branches per PB	10.67±1.37	8.43±0.55	3.46	0.0072
PB length (mm)	9.51±0.90	11.72±1.14	-3.08	0.0218
PB diameter (mm)	8.09±0.47	8.95±0.60	-2.28	0.0629
No. of fertile seeds per PB	52.87±6.98	37.56±4.30	3.37	0.0150
No. of seeds per PB	54.93±7.73	39.67±3.46	3.16	0.0196
Seeds setting rate	97.00±2.12	95.95±2.76	0.61	0.5653
Grain length(mm)	1.76±0.10	1.94±0.11	4.94	<0.0001
Grain diameter(mm)	1.63±0.05	1.72±0.03	6.10	<0.0001
Hulled grain length(mm)	1.40±0.08	1.45±0.09	1.77	0.0870
Hulled grain diameter(mm)	1.55±0.04	1.62±0.05	-4.37	0.0002

### The *lp1* mutation also affects plant height and seed size in foxtail millet

**Fig 2:**
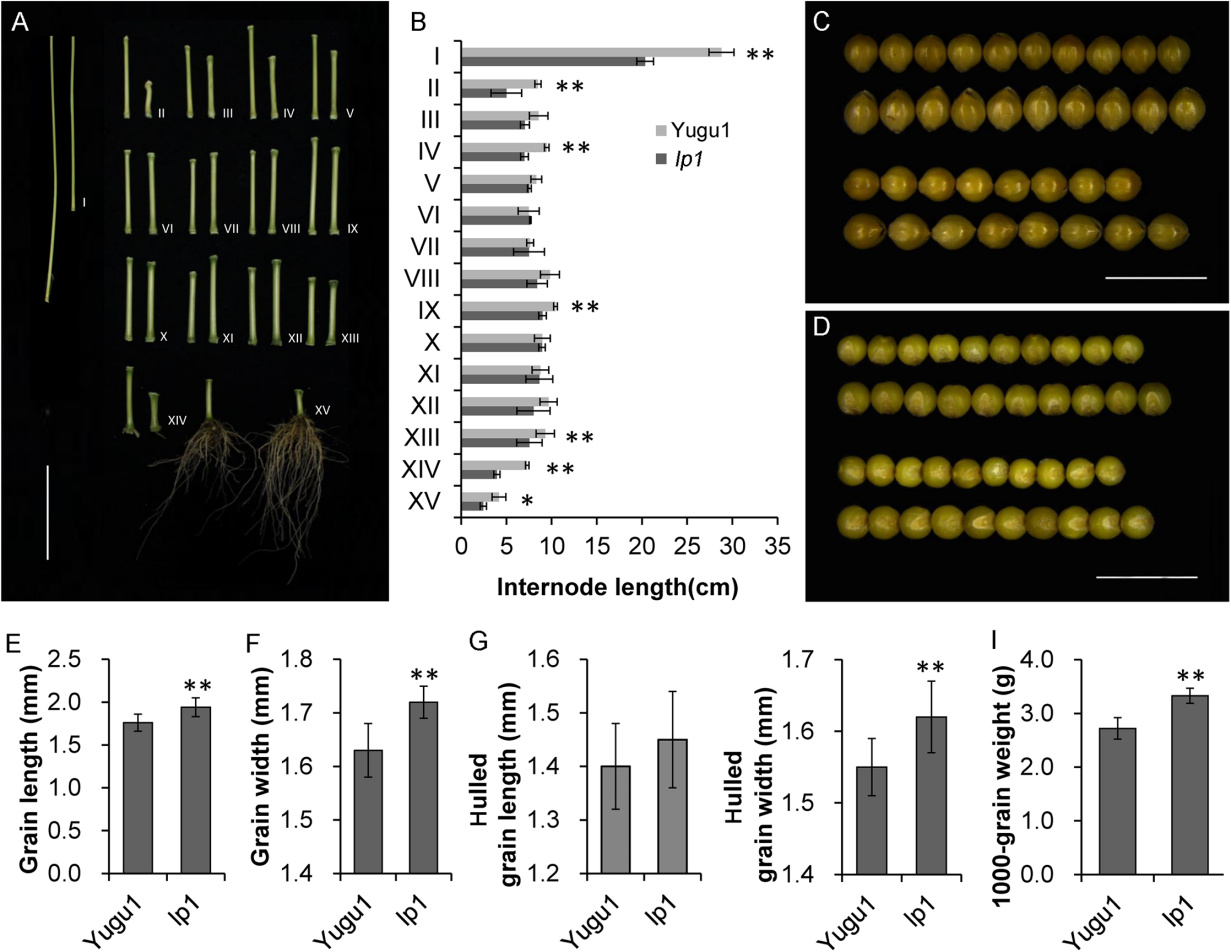
Comparisons of plant height and grain size between the wild-type ‘Yugu1’ and the *lp1* mutant. (A) Internodes and roots of ‘Yugu1’ (left) and *lp1* (right) at the mature stage, bar = 10 cm. I–XV indicate the internode numbers under the panicle. (B) Comparison of internode lengths between the mutant and wild-type. Welch’s two-sample t-test, n = 3 biological replications, * indicates P < 0.05, ** indicates P < 0.01. (C) Morphological comparison of unhulled grain. The first and second rows represent the unhulled grain width of ‘Yugu1’ (up) and *lp1* (down), while the third and fourth rows represent the unhulled grain lengths of ‘Yugu1’ and *lp1*, bar = 5 mm. (D) Morphological comparison of hulled grain. The first and second rows represent the hulled grain widths, while the third and fourth rows represent the hulled grain lengths of ‘Yugu1’ and *lp1*, bar = 5 mm. (E–I) Statistics of grain length, grain width, hulled grain length, hulled grain width, and 1,000-grain weight in ‘Yugu1’ and *lp1*. Welch’s two-sample t-test, n = 10 biological replications, asterisks indicate P < 0.01.

The average plant height of *lp1* was less than that of ‘Yugu1’ (Fig 1A). The reductions in the upper and lower elongated internode lengths were statistically significant, while the lengths of the middle internodes were only slightly change (Fig 2A and 2B). The *lp1* mutation produced larger grains by increasing the seed lengths and widths (Fig 2C–2H), and ultimately increased the average 1,000-grain weight by 22.4% (Fig 2I). However, because of the reductions in primary branch numbers and grain numbers per branch, the overall grain yield per plant significantly decreased (Table 1).

### Map-based cloning combined with BSA sequencing identified the causal gene *LP1*

To identify the causal gene, *lp1* was hybridized with another landrace ‘SSR41’, resulting in the F_2_ mapping population. All of the individuals in the F_1_ generation exhibited the normal panicle phenotype. The F_2_ generation contained 1,003 individuals, 769 were phenotypically wild-type and 234 were homozygous *lp1* plants. The segregation ratio of the F_2_ population was 3:1 (χ^2^ = 1.40 < χ^2^_0.05_ = 3.84), which fit the Mendelian segregation, suggesting that the loose panicle phenotype of *lp1* was controlled by a single recessive nuclear gene.

**Fig 3:**
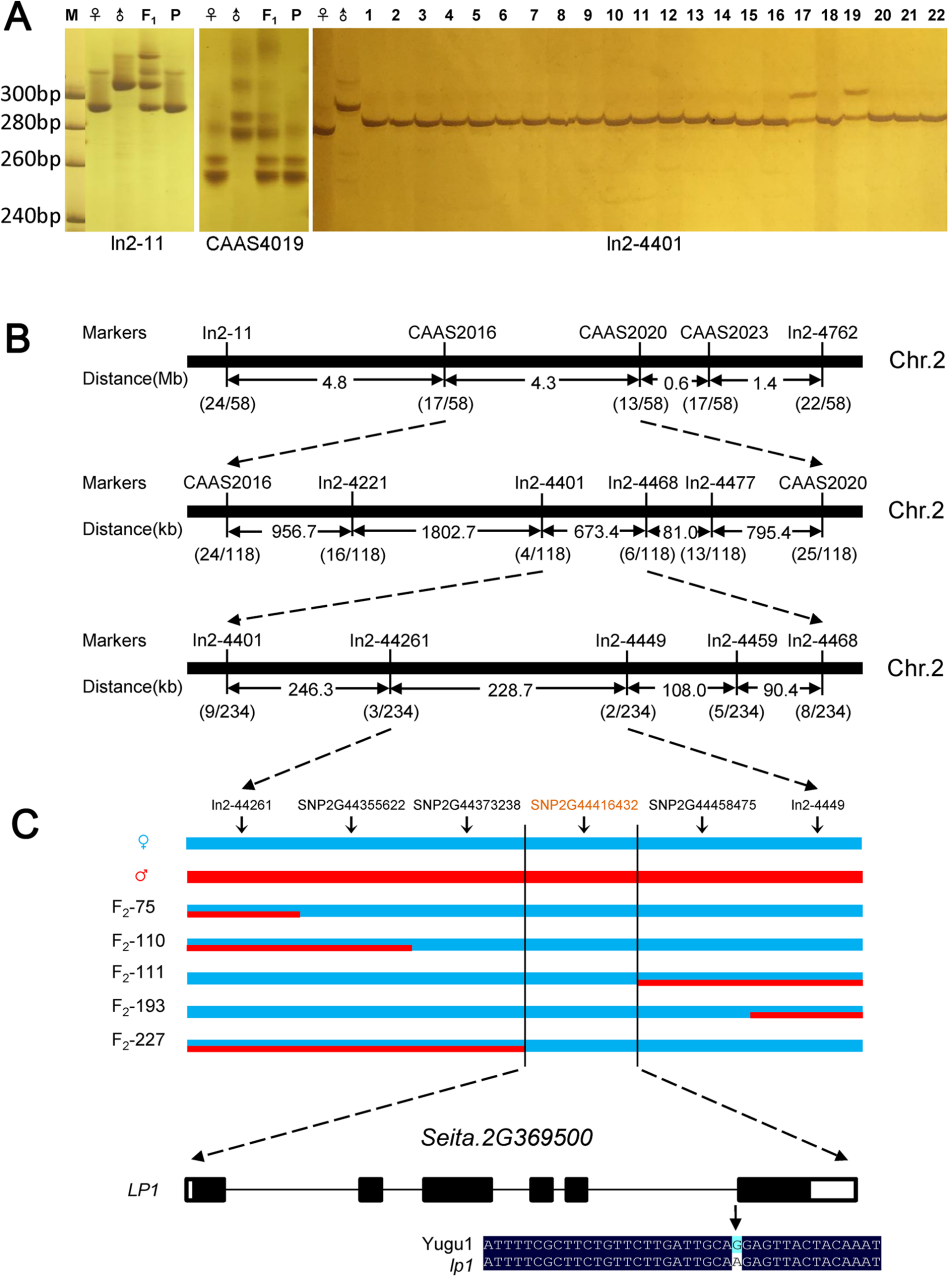
Identification of the LP1 locus. (A) Examples of the molecular markers used for map-based cloning. CAAS4019, In2-11, and In2-4401 are SSR and indel markers that are closely linked to the LP1 locus. M, molecular weight marker; ♀, female parents (*lp1*); ♂, male parents (‘SSR41’); F_1_, F_1_ generation from *lp1* × ‘SSR41’ cross; P, DNA pools of recessive homozygous individual in F_2_ population; 1–22, different recessive homozygous individuals. (B) Fine mapping of the *LP1* gene using molecular markers. (C) Co-segregation analysis of the BC_1_F_2_ population using dCAPS markers; ♀, female parents (*lp1*); ♂, male parents (‘Yugu1’).

For map-based cloning, we selected 45 molecular markers that were evenly distributed on all nine chromosomes of *S*. *italica*. A bulked segregation analysis showed that the causal gene was closely linked with the molecular markers ln2-1 and CAAS4019 (Fig 3A), indicating that *LP1* is located at the end of chromosome 2. In total, 234 F_2_ homozygous recessive individuals were used for fine mapping. LP1 was mapped to a 673.4-kb region between markers ln2-4401 and ln2-4468. To narrow the mapping intervals, four new indel markers were designed. Finally, the causal gene was limited to a 228.7-kb region between the markers ln2-44261 and ln2-4449 (Fig 3B).

To determine the candidate gene, we employed MutMap, a gene isolation method based on high-throughput genome resequencing of pooled DNA [28]. The *lp1* plants (♀) were backcrossed with wild-type ‘Yugu1’ (♂) to construct the BC_1_F_2_ population. Bulk genomic DNA samples extracted from 30 BC_1_F_2_ individuals exhibiting the mutant phenotype were used for whole genome resequencing. Using MutMap analysis, we obtained the SNP index for each reliable SNP, and we plotted SNP indices for all 9 chromosomes of foxtail millet (S1 Fig). The result showed that there was a peak around 44 Mb region on Chromosome 2. Combine with our map-based cloning result (Fig 3A and 3B), we identified a single unique genomic region (Chr. 2: 44,261,991–44,490,709 bp) harboring the causal SNP. In total, 18 SNPs between mutant and wild-type plants were located inside the mapping interval. Four homozygous SNPs (SNP-index = 1) were then selected because the mutant phenotype of *lp1* was controlled by a single recessive nuclear gene. We further examined 297 homozygous recessive individuals from the BC_1_F_2_ population for the presence of the four SNPs. Only the SNP (G to A) at 44,416,432 bp on chromosome 2 co-segregated with the loose panicle phenotype (Fig 3C). According to the *S*. *italica* v2.2 genome project (http://phytozome.jgi.doe.gov), the causal SNP (SNP2G44416432) was located inside the genomic region of *Seita*.*2G369500*, suggesting that *Seita*.*2G369500* corresponds to *LP1*, which is responsible for the mutant phenotype.

### *LP1* encodes a novel WRKY transcription factor in *S*. *italica*

**Fig 4:**
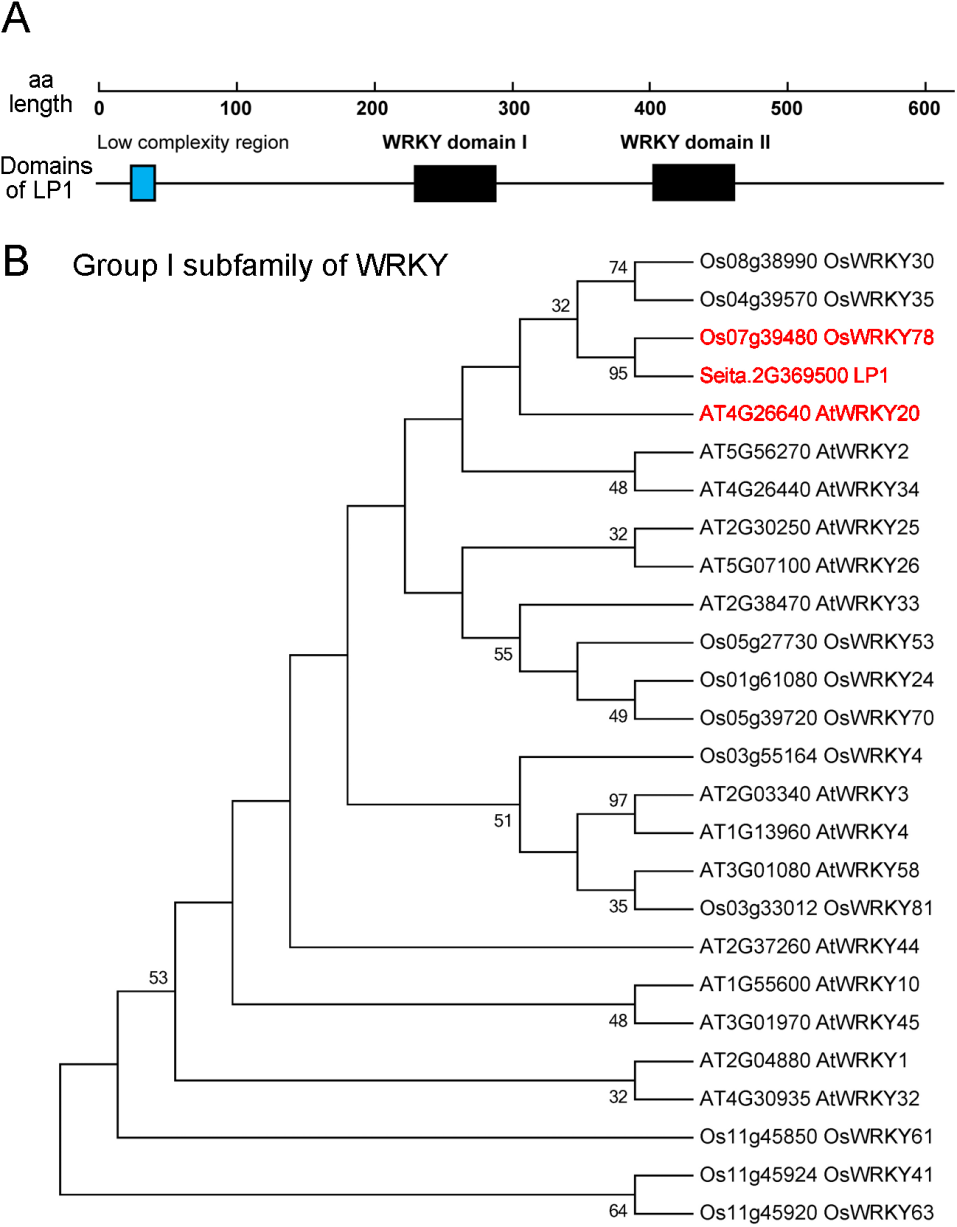
Conserved features of LP1 and a phylogenetic analysis. (A) Conserved domains of the LP1 protein. LP1 has two conserved WRKY domains. (B) Phylogenetic analysis of LP1 and its homologs in *Arabidopsis* and rice revealed that LP1 belongs to Group I of the WRKY subfamily. The red letters stand for SiLP1 and its two closest homologous proteins in *O*. *sativa* (OsWRKY78) and *Arabidopsis* (AtWRKY20). Phylogenetic tree was constructed using the deduced full-length protein sequences of LP1 and other WRKYs selected from previous researches [31, 32]. MEGA5 software (www.megasoftware.net) was employed with the maximum likelihood method, JTT model and 1000 bootstrap replicates. The bootstrap value for each node is shown in the figure.

A BLAST algorithm based search against the *S*. *italica* genome database revealed that *LP1* is a single-copy gene with a coding sequence length of 1,842 bp. The LP1 peptide sequence contains 613 amino acid residues, with a predicted molecular mass of 65.75 kDa and an isoelectric point of 6.59. Based on the protein domain and functional site analyses, the LP1 protein was identified as a member of the WRKY transcription factor superfamily, and it contained two typical conserved WRKY domains (IPR003657) and a low complexity region (Fig 4A). The WRKY proteins in higher plants can be divided into five subfamilies, Groups I, IIa + IIb, IIc, IId + IIe, and III [20]. A phylogenetic analysis was carried out using the LP1 sequence and previously characterized *WRKY* genes in *Arabidopsis* [31] and *O*. *sativa* [32]. Besides, Muthamilarasan et al. (2015) identified 105 WRKY proteins in *S*.*italica* genome via bioinformatics analysis [21]. After comparing our result with previous studies, we found that LP1 was classified into WRKY subfamily Group I (Fig 4B). Another WRKY family member, OsWRKY78, classified into the same group as LP1 was determined to regulate plant height and reproductive organ development in *O*. *sativa*, which is similar to *S*. *italica*, indicating that this type of *WRKY* gene may have shared functions across species.

### A single nucleotide variation caused the alternative splicing of *LP1*, which eventually affected the WRKY domain’s structure in the mutant

**Fig 5:**
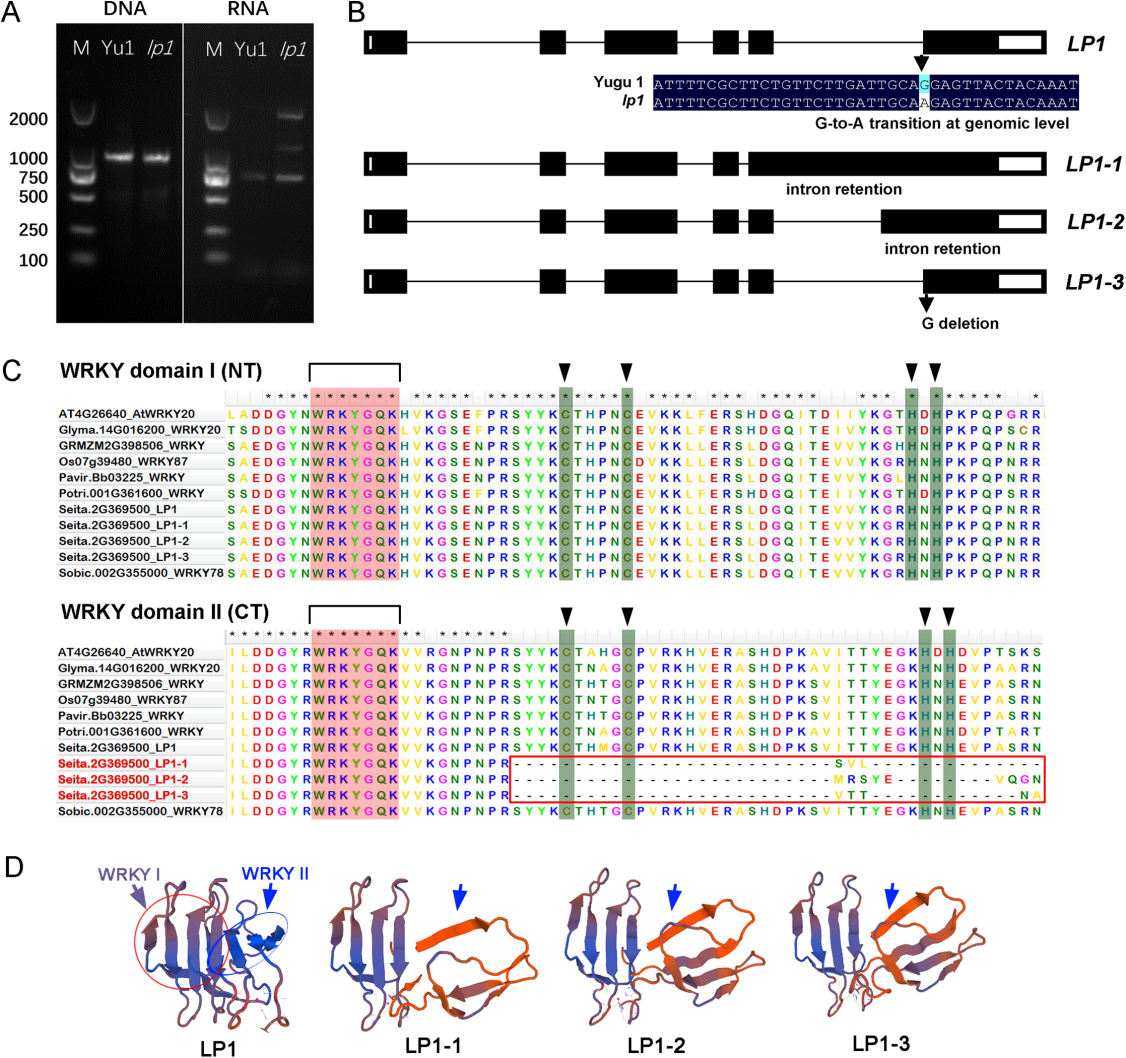
Characterization of *LP1* in wild-type ‘Yugu1’ and the *lp1* mutant. (A) PCR analysis of *LP1* at the genomic DNA and RNA levels. Different bands were detected in the *lp1* mutant at the RNA level when compared to wild-type ‘Yugu1’. (B) Comparison of the gene structures of *LP1* alleles. Compared with the *LP1* sequence in wild-type plants, a single G to A mutation was found in the mutant, which led to the production of three different splice variants (*LP1-1*, *LP1-2*, and *LP1-3*) at the transcriptomic level. (C) Comparison of WRKY domain sequences among different species. WRKY domain I (NT) indicates the first WRKY domain at the N-terminus of LP1. WRKY domain II (CT) indicates the second WRKY domain at the C-terminus of LP1. The C2H2 motif sequence in WRKY II was completely lost in all three LP1 variants in the mutant. (D) Comparison of the WRKY domain structure in ‘Yugu1’ and *lp1*. *In silico* structural modeling of the LP1 protein was performed by SWISS-MODEL (swissmodel.expasy.org). The WRKY I domain of LP1 was not affected, while the WRKY II domain structure were severely disrupted in the *lp1* mutant.

Sanger sequencing of the full-length genomic DNA sequences of *LP1* in both mutants and wild-type plants showed that only a single nucleotide G-to-A transition at position 4,151 (from initiation codon) was detected in the fifth intron of *Seita*.*2G369500*. The mutation break the GT-AG rule (original GT-AG is changed in to GT-AA), which would cause abnormal mRNA splice events in mutant plants. As expected, RT-PCR and gel electrophoresis analyses showed that there were three new putative splice variants in *lp1* at the RNA level that are different from the original sequence in wild-type plants (Fig 5A). To verify the existence of *LP1* alternative splicing events, we sequenced 20 cDNA clones from *lp1* and wild-type ‘Yugu1’. Sequencing indicated that there are three different *LP1* transcripts in the mutant, while only one transcript type existed in the wild-type control. The first and the second variants of LP1 (named as LP1-1 and LP1-2) were produced by intron retention. As shown in Fig 5B, the whole fifth intron was retained in the coding region of LP1-1, while for LP1-2, a 340-bp sequence from 3′ end of the fifth intron was retained. In *LP1-3*, a single nucleotide deletion (G deletion) occurred at the start position of the sixth exon. All three types of mutation led to the destruction of the C2H2 zinc-finger structure at the C-terminus of the second WRKY domain, but they did not affect the first WRKY domain (Fig 5C). Protein tertiary structure modeling also showed that the N-terminal WRKY domain remained unchanged in LP1-1, LP1-2, and LP1-3, while the C-terminal WRKY domain was severely affected in all three LP1 isoforms in the mutant (Fig 5D).

### Expression pattern of *S*. *italica LP1*

**Fig 6:**
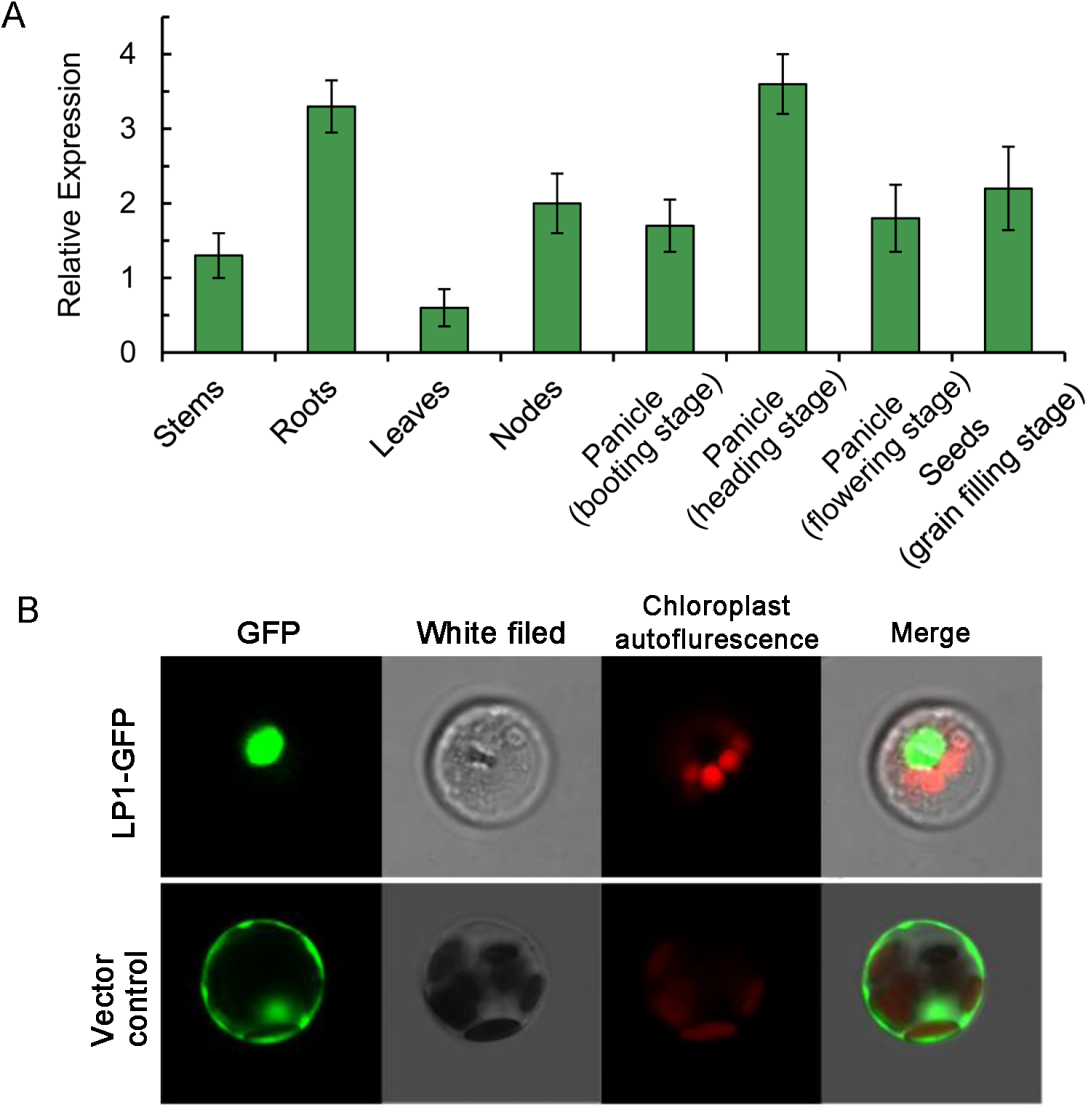
Expression pattern of *LP1*. (A) Quantitative real-time PCR analysis of *LP1* expression levels in different foxtail millet organs. Mean expression levels and standard deviations were calculated from three independent biological replications. (B) Subcellular localization of LP1 protein. The LP1-GFP fusion protein was constructed and expressed in foxtail millet mesophyll protoplasts.

To study the tissue-specific expression pattern of *LP1* in foxtail millet, we examined its gene expression levels in different tissues at various developmental stages. As shown in Fig 6A, *LP1* can be detected in all of the organs we collected, including leaves, stems, nodes, and panicles at different developmental stages. However, *LP1* was relatively highly expressed in panicles at the booting stage, in roots, and in seeds at the grain-filling stage. For vegetative organs, *LP1* was expressed more highly in nodes and elongation stems than in leaves. This expression pattern is consistent with its gene function in regulating panicle shape, plant height, and seed size. The subcellular localization of *LP1* clearly showed that the LP1-GFP fusion protein predominantly accumulated in the nuclei of foxtail millet protoplasts, which confirmed its role as a transcription factor (Fig 6B).

## Discussion

### *Setaria* is a feasible and efficient model system for gene mapping

*Setaria* spp. (mainly referring to *S*. *italica* and *S*. *viridis*) have recently been proposed as model systems in studies of C_4_ photosynthesis, abiotic stress tolerance, and bioenergy feed stocks [1, 2]. The availability of complete high quality *Setaria* genomes [33, 34] and abundant polymorphic molecular markers [26, 27], as well as the development of tissue culture and genetic transformation methods [1], have been rapidly pushing these studies. Moreover, our previous researches [4–6] evidenced that forward-genetics-based gene mapping and function analysis systems can be applied with high efficiency in foxtail millet just as in the classical model plants *Arabidopsis* and rice.

MutMap was recently developed in rice based on high-throughput genome resequencing techniques [28]. In this trial, we employed a MutMap analysis using the bulked DNA of recessive individuals derived from backcrosses between the *S*. *italica* EMS-induced mutant *lp1* and wild-type ‘Yugu1’. A novel transcription factor *LP1*, responsible for the *lp1* mutant phenotypes, was identified through a map-based cloning and high-throughput genome sequencing integrated method. Less than 300 recessive F_2_ individuals and 53 molecular markers were used to determine the causal gene. We identified that *LP1*, which belongs to WRKY superfamily Group I, played important roles in regulating panicle shape, plant height, and seed development in *Setaria*. This reinforces that *S*. *italic* is a feasible and efficient model plant for locus mapping and gene function analyses.

### Role of *LP1* in regulating panicle development, stem elongation, and seed size in foxtail millet

Panicle development is a main trait that determines the yields of many crop plants. Molecular cloning and functional analyses have identified a number of genes associated with panicle development in rice, such as *LAX1/2* [9, 10], *DEP1* [15], *SP1* [35], and *ASP1* [36]. WRKY proteins are a large transcription factor family regulating various biological processes in higher plants. A majority of researches on WRKY transcription factors have been related to biotic or abiotic stress responses [19]. Although, there have been a few reports that elucidated their roles in seed development [23] and leaf senescence [25]. However, few *WRKY* genes have been reported to regulate panicle development. The *LP1* gene, which encodes a novel WRKY transcription factor, directly regulated panicle development in foxtail millet. Subcellular localization indicated that *LP1* localized in the nucleus, which coincided with the features of the transcription factor. *LP1* was expressed more highly in young inflorescence and seeds compared with in other tissues, which confirmed its role in regulating panicle development and seed size.

The *lp1* mutant, isolated from a large EMS-induced *S*. *italica* mutant library, exhibited a sparse and loose panicle phenotype (Fig 1). In *O*. *sativa*, two lax panicle mutants *lax1* [9] and *lax2* [10], also display phenotypes similar to those of the *S*. *italica lp1* mutant. However, there are still some differences between *lp1* and *lax1*/*lax2*. First, *LAX1* encodes a bHLH transcription factor that may control axillary meristem initiation through the auxin pathway. The loss of LAX1/2 function led to a dramatically reduced number of secondary branches (−62.1% compared with wild-type) and spikelets in mutants, but did not affect primary branches in rice. However, in the foxtail millet *lp1* mutant, both primary and secondary branches decreased (−10.4% and −21.0%, respectively). Additionally, the panicle length of *lp1* was longer than that of wild-type, which is opposite that of *lax1*/*lax2*. In addition, *lp1* mutants also influenced stem elongation and seed size, while no significant differences were found in plant height and seed development in rice *lax* mutants. Further research should be undertaken to elucidate the possible LP1-mediated pathways and genetic networks associated with these characterized phenotypes in foxtail millet.

### A comparative analysis revealed common aspects and differences in the effects of *LP1* and its homologs

The *lp1* mutation was controlled by a single recessive WRKY transcription factor (LP1). Although studies related to WRKYs are limited in *S*. *italica*, *LP1*’s closest homologs have been identified in other model plants, including *Arabidopsis* (*AtWRKY20* [37, 38]), and rice (*OsWRKY78* [21]). For *Arabidopsis WRKY20*, studies were mainly concentrated on its role under biotic/abiotic stress conditions [35, 36]. Using large-scale microarray and qPCR data, Luo et al. (2013) determined that the homolog of *AtWRKY20* in wild soybean (GsWRKY20) was significantly up-regulated under abscisic acid (ABA), salt, and drought stresses. The overexpression of *GsWRKY20* in *Arabidopsis* resulted in changes ABA sensitivity and enhanced drought tolerance in transgenic lines [35]. Although there have been studies that confirmed the roles of *WRKY20* under various stresses, limited research suggested that the homologous genes of *LP1* in dicots could regulate organ development. Whether this is the result of the gene’s functional diversification between monocots and dicots, or that the *LP1* function in regulating development has not yet been characterized in dicots, remains unknown.

Unlike the role that *WRKY20* plays in *Arabidopsis*, foxtail millet *LP1* and rice *WRKY78* have similar functions in regulating plant development. In rice, *OsWRKY78* RNAi transgenic plants exhibit semi-dwarfism and sheathed panicles [21]. Similar phenotypes were observed in foxtail millet *lp1*, which is also a semi-dwarf mutant having a significant decrease in the length of the uppermost internode below the panicle (Fig 2). The major difference between *lp1* and *OsWRKY78* RNAi plants involves their influences on seed development. The seeds from rice RNAi plants were smaller than those of the wild-type control [21], but in foxtail millet *lp1*, the lengths and widths of the seeds, and the 1,000-grain weight, increased. Although both rice and foxtail millet are grass species, *O*. *sativa* is a kind of Ehrhartoideae crop, while *S*. *italica*/*S*. *viridis* belongs to Panicoideae. Thus, *LP1* gene functional diversification may exist between Panicoideae and Ehrhartoideae. Additionally, there is a balance between seed size and panicle density [39]. The increased seed size of *lp1* may result from the increase in panicle length and lower number of seeds per branch.

In summary, our study characterized a loose panicle *S*. *italica* mutant having pleiotropic effects on plant development. A novel WRKY transcription factor, *LP1*, was identified to be the causal gene. The putative function and expression pattern of *LP1* were preliminarily characterized. Our research provided useful information on the molecular mechanism of the WRKY transcription factor involved in regulating plant height, panicle morphogenesis, and seed development, and *Setaria* was shown to be an efficient model for functional genomics studies. Future research is needed to identify the downstream targets of *LP1* and elucidate the transcriptional regulatory networks that are associated with panicle development in foxtail millet.

## Supporting information

S1 Table Primers designed for map-based cloning, RT-PCR, and qPCR. (DOCX)

S1 Fig SNP index plots for MutMap analysis. (TIF)
