# Supplementary material for: Loose Panicle1 encoding a novel WRKY transcription factor, regulates panicle development, stem elongation, and seed size in foxtail millet [Setaria italica (L.) P. Beauv.]
Source: PLoS One. 2017 Jun 1;12(6):e0178730. doi: 10.1371/journal.pone.0178730 (PMC5453597; doi:10.1371/journal.pone.0178730)
Supplement: S1 Table — (DOCX) [file pone.0178730.s001.docx]

S1 Table. Primers designed for map-based cloning, RT-PCR, and qPCR.

| No. | Name | Forward sequence (5’-3’) | Reverse sequence (5’-3’) |
| --- | --- | --- | --- |
| 1 | In2-11 | CTGCCCACTTGCAACTGAAC | TGCTTAGCCAAGTTCTCGCA |
| 2 | CAAS4019 | acttccgggttgccggtgatgttcct | tccactttccccatccttctccgccgt |
| 3 | In2-4401 | AACTTGCTTGGGACT | ATAATGCGTTTGGTG |
| 4 | CAAS2016 | ttgcgctgttgcgatctgccgctct | atggcgtccattcctctccgctggt |
| 5 | CAAS2020 | tcgtagcacccactgcacgctccta | ctcacctgttcccgaatcaacctgccg |
| 6 | CAAS2023 | cggccgcggcaggcatgattgattgat | aggccctgcatccagctcagcacat |
| 7 | In2-4762 | CTGACCACTCCGCTTCT | GATTCGTCTCGCAAAAT |
| 8 | In2-4221 | AAGGTATCTGATGGCAAAG | CACTCCACCGCAACG |
| 9 | In2-4468 | ATGGGAGGGAGATGAATGG | TTGGTGGTCGTGGTGGG |
| 10 | In2-4477 | GCAAGCTGGTACTGTGG | TGTTGGCGATGGGTG |
| 11 | In2-44261 | ATTCTTTCAGCCTCAGTCG | TGTCCTAATCTCGGATCTTT |
| 12 | In2-4449 | CCGCAGATAGAATAAACG | TAGCACCACATTGTCAAAT |
| 13 | In2-4459 | TGTAGCACCACATTATCAA | TTTAGCAGTGCCGTGT |
| 14 | SNP2G44355622 | CGAGCCATTGTCCTG | CCACTCGTTACCACCA |
| 15 | SNP2G44373238 | CACGGGAATAGAACTCG | GTAAAGATGTATGGTGGAAA |
| 16 | SNP2G44416432 | TGGGTGTTGAGGTATGAC | TGGAAGGACCTGTTGC |
| 17 | SNP2G44458475 | TGAACTGCAAGTGATT | GAGATGTTTTGGTTTG |
| 18 | LP1_DNA | CAAGAATTAGAATAGTGGGAGA | TGCGTCGCTGTGGTT |
| 19 | LP1_RNA | AGAGGATGGCTATAACTGG | TCGGCTGCCTGAGAA |
| 20 | Actin | TATGGGTCATCAACAGCTTGTC | GTAGTCCCTCGTGATGAGATCC |
| 21 | qPCR2G369500 | GTGCTGGTAACTTAGTGATG | ATGACGACGGTGATATGT |
