# Supplementary figures and images for: Loose Panicle1 encoding a novel WRKY transcription factor, regulates panicle development, stem elongation, and seed size in foxtail millet [Setaria italica (L.) P. Beauv.]
Source: PLoS One. 2017 Jun 1;12(6):e0178730. doi: 10.1371/journal.pone.0178730 (PMC5453597; doi:10.1371/journal.pone.0178730)

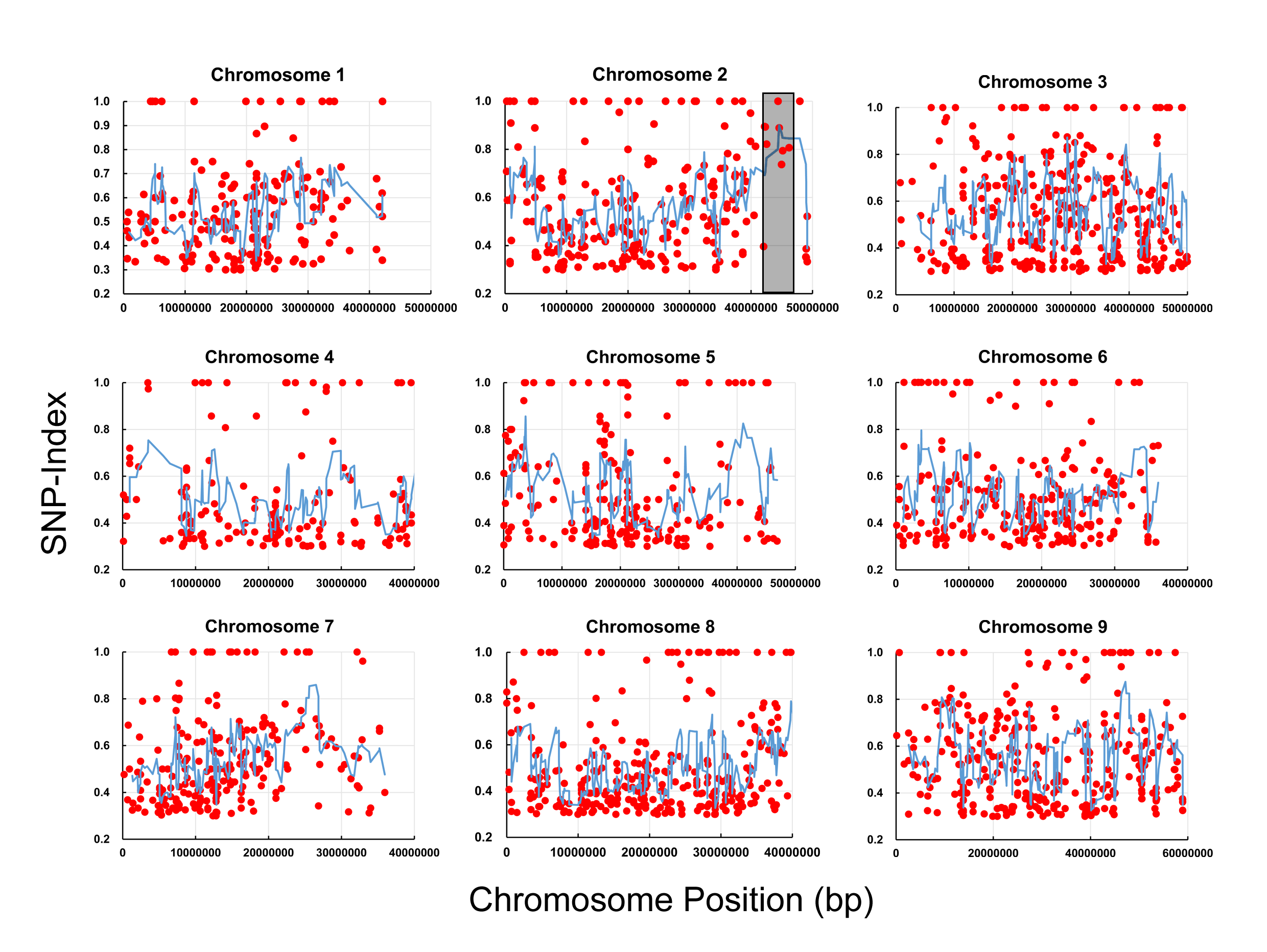

Supplement: S1 Fig — (TIF) [file pone.0178730.s002.tif]
